# Strongly Coupled Plasmon Polaritons in Gold and Epsilon-Near-Zero Bifilms

**DOI:** 10.1021/acsphotonics.2c01412

**Published:** 2023-01-03

**Authors:** Saumya Choudhary, Saleem Iqbal, Mohammad Karimi, Orad Reshef, M. Zahirul Alam, Robert W. Boyd

**Affiliations:** † Institute of Optics, University of Rochester, Rochester, New York14627, United States; ‡ Department of Physics, University of Ottawa, Ottawa, OntarioK1N 6N5, Canada

**Keywords:** surface plasmon polaritons, epsilon-near-zero, strong coupling, hybridization, field enhancement, nanophotonics

## Abstract

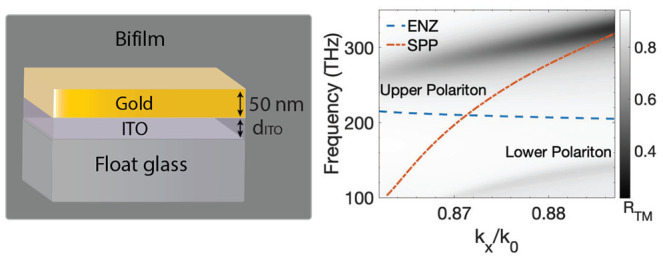

Epsilon-near-zero (ENZ) polaritons in a thin transparent conducting-oxide film exhibit a significant electric field enhancement and localization within the film at frequencies close to their plasma frequency, but do not propagate. Meanwhile, plasmon polariton modes in thin metallic films can propagate for several microns, but are more loosely confined in the metal. Here, we propose a strongly coupled bilayered structure of a thin gold film on a thin indium tin oxide (ITO) film that supports hybrid polariton modes. We experimentally characterize the dispersion of these modes and show that they have propagation lengths of 4–8 μm while retaining mode confinement greater than that of the polariton in gold films by nearly an order of magnitude. We study the tunability of this coupling strength by varying the thickness of the ITO film and show that ultrastrong coupling is possible at certain thicknesses. The unusual linear and nonlinear optical properties of ITO at ENZ frequencies make these bifilms useful for the active tuning of strong coupling, ultrafast switching, and enhanced nonlinear interactions at near-infrared frequencies.

## Introduction

Two harmonic oscillators become strongly coupled when they exchange energy faster than the rate at which energy decays from the system. The coupled system has eigenstates that are a hybrid of those of the two uncoupled oscillators and which show a characteristic avoided crossing of their dispersion lines around the degeneracy point of the uncoupled oscillators.^1,2^ Strong coupling between dipolar oscillators and a cavity has been achieved previously either by reducing the cavity mode volume or by enhancing the oscillator strength.^2−10^ Surface plasmon polaritons (SPP) supported by a metal–dielectric interface also have small mode volumes that make them excellent candidates for strong coupling to other localized modes.^2,11,12^ SPPs are formed by strong coupling between light and the charge density oscillations in the metal, and they have a large field confinement along the metal–dielectric interface.^13,14^ A specific type of SPP mode, called a long-range surface plasmon polariton (LR-SPP), is supported by thin metallic films and can propagate for hundreds of microns.^13−15^

Thin films of transparent conducting oxides (TCO), such as indium tin oxide (ITO), also support polaritons. Close to their plasma frequency, the permittivity of these oxides vanishes, a condition also referred to as “epsilon-near-zero” (ENZ).^16,17^ The LR-SPP mode of very thin TCO films is modified such that it has a very large and localized longitudinal field component within the film and, unlike the highly dispersive LR-SPP mode in metallic films, has a flat dispersion line, rendering it nonpropagative. This special mode is referred to as the “ENZ” mode.^18^ It is a collective excitation of free electrons in the TCO film that is strongly absorptive. There is also considerable recent interest in the unusual linear and nonlinear optical phenomena in the ENZ regime,^17,19^ including giant nonlinear optical response^16,20−27^ due in part to the relaxation of phase-matching constraints^28,29^ and large field enhancements.^20,22^ However, the large absorption losses associated with most ENZ materials limit their effective interactions lengths to subwavelength scales. Metamaterial resonators strongly coupled to the ENZ mode of TCO and other doped semiconductor films can enhance the nonlinear response through local field enhancement.^30−33^ However, these coupled systems are still limited by their subwavelength interaction lengths. Hence, it is interesting to explore structures that support hybrid modes formed by strong coupling between the ENZ mode and guided modes, such as polaritons. Previous demonstrations of strong coupling between polaritons and the ENZ mode have been performed with phonon polaritons,^34^ and with plasmon polaritons^35^ at mid-infrared frequencies.

**Figure 1:**
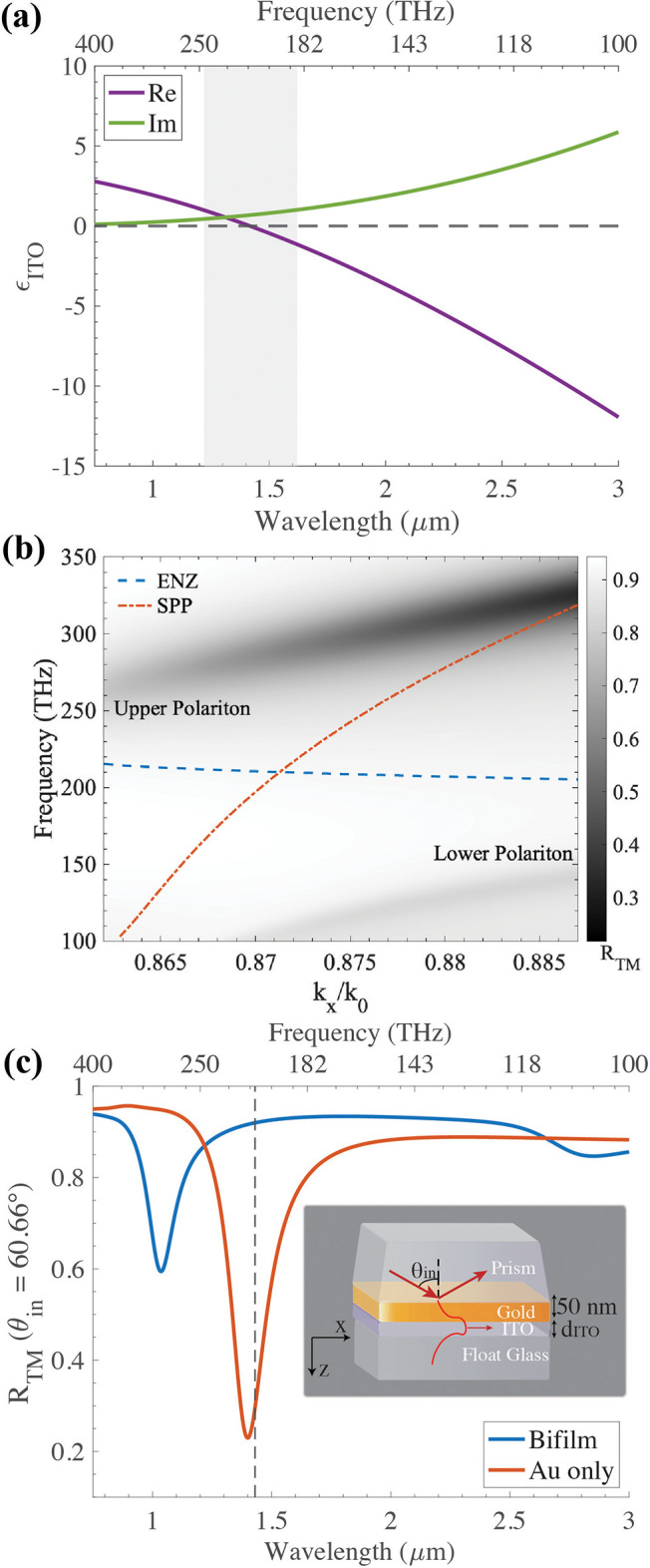
(a) Permittivity spectrum of a representative ITO sample. The grayed region shows the absorption band of the ENZ mode. (b) Simulated reflectance map of TM-polarized light (*R*_TM_) for a bifilm (inset in (c)) with a 23-nm-thick film of the same ITO plotted against normalized wavevector (*k*_N_ = *k*_*x*_/*k*_0_; *k*_0_ is the propagation constant within the N-SF11 prism used for coupling) and frequency axes. The dispersion lines of the SPP mode (red, solid) and the ENZ mode (dashed, blue) are overlaid. (c) Linecut (blue) of the *R*_TM_ map in (b) and the *R*_TM_ spectrum of a standalone gold film (red) at an angle of incidence θ_in_ close to crossing of the SPP and the ENZ dispersion lines.

We propose a bifilm structure consisting of a gold film deposited on a thin ITO film backed by a float glass substrate. This structure supports guided modes at near-infrared (NIR) frequencies. Since the plasma frequency of gold lies in the ultraviolet region, the dispersion lines of the LR-SPP mode in the gold film (the red dot-dashed line in Figure 1(b)) and the ENZ mode in the ITO film (the blue dashed line in Figure 1(b)) cross around the ENZ region of ITO, which occurs at NIR frequencies. When placed in spatial proximity as in the bifilm structure (the inset in Figure 1(c)), these constituent modes couple strongly in this ENZ region with a strength dependent on their spatial overlap. The two hybrid modes thus formed have dispersion lines that show avoided (or anti) crossing, where they have at least an order of magnitude larger confinement in the ITO film than the LR-SPP mode in the gold film. Also, unlike the ENZ mode, they can propagate for several microns because of significantly lower losses. Further, we examine the dependence of coupling strength of the constituent modes on the thickness of the ITO film and show that ultrastrong coupling, wherein their coupling strength becomes comparable to the anticrossing frequency,^36,37^ can be achieved at certain thicknesses.

## Results and Discussion

Figure 1(b) shows the reflectance map *R*_TM_ of TM (or p)-polarized light obtained from transfer matrix method (TMM) simulations of a bifilm made of a 50-nm-thick layer of gold and a 23-nm-thick layer of ITO, whose permittivity (ϵ_ITO_) spectrum is shown in Figure 1(a). The permittivity of gold determined by Johnson and Christy^38^ is used for all the calculations performed here. We use the Kretschmann–Raethar configuration,^13^ shown in the inset of Figure 1(c), to excite polaritons along the gold–ITO interface through a high-index N-SF11 prism kept in contact with the gold facet of the sample using an index-matching oil. The dispersion of the coupling prism is excluded in Figure 1(b) by plotting the reflectance spectra in the normalized wavevector (*k*_*x*_/*k*_0_) and frequency (ν) space. The dispersion lines of the LR-SPP mode (red, dot-dashed) and the ENZ mode (blue, dashed) are obtained from the locus of minima of the reflectance map of a standalone gold film and a standalone ITO film, respectively. The LR-SPP (just called SPP from now on) mode has a strongly wavevector dependent dispersion, while the ENZ mode has a flat dispersion pinned at the ENZ frequency. The two distinct branches of minima in the reflectance map correspond to the two hybrid modes of the bifilm, and they asymptotically approach the dispersion lines of the constituent SPP and ENZ modes away from their avoided crossing point.

**Figure 2:**
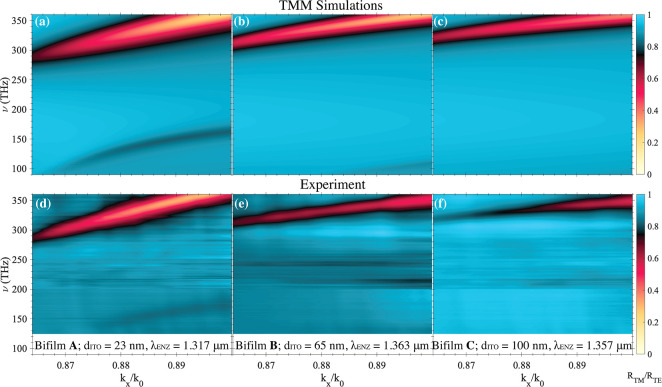
Simulated (top) and measured (bottom) reflectance maps of the three bifilm samples in the *k*_*x*_/*k*_0_–ν space. The TM-polarized reflectance spectra *R*_TM_ are normalized to the TE-polarized spectra *R*_TE_ at each incident wavevector to exclude measurement artifacts. The range of wavevectors is limited by the critical angle for the prism–substrate interface, and the maximum rotation of the prism–sample assembly is possible without clipping the incoming field.

We experimentally characterize the dispersion of bifilms through attenuated total reflection spectroscopy measurements. Figure 2(d)–(f) show the measured and Figure 2(a)–(c) the simulated reflectance maps of three bifilm samples A, B, and C, each with a 50-nm-thick gold film and ITO films with thicknesses (*d*_ITO_) of 23, 65, and 100 nm, respectively. The three ITO films have similar properties with their ENZ wavelengths at 1.317, 1.363, and 1.357 μm, respectively. See Supplementary Section S1 for their permittivity spectra, and S2 for the experimental details. We observe both the high-frequency (upper) and the low-frequency (lower) polariton branches in the measured (Figure 2(d)) and the simulated (Figure 2(a)) reflectance maps of the thinnest bifilm (A). The simulated maps of bifilms B and C show that the spectral separation between the two polariton branches, henceforth referred to as the “polariton band gap”, increases with *d*_ITO_.

As an aside, we note that polaritons are said to be critically coupled when the coupling losses are balanced by the absorption losses.^13^ The polariton band gap for all three bifilms is large enough that while the upper polariton is coupled efficiently and has a prominent resonance dip, the lower polariton with its comparatively smaller resonance dip is not coupled as efficiently. This inefficient coupling and large absorption losses of the lower polariton contribute to its visibility being smaller than the upper polariton. For bifilm C, the lower polariton branch is not visible in Figure 2(c). However, it becomes more prominent if the absorption losses within ITO are reduced. See Section S4 in the Supporting Information for more details. The measured reflectance maps of bifilms B and C do not show the lower polariton branch, as we are limited by the spectral range of our spectrometers and white light source. However, the measured and the simulated maps for all three bifilms are in reasonable agreement in the spectral range shown here.

For an insight into the formation of these hybrid polaritons, we analytically model the bifilm as a system of two coupled harmonic oscillators that describe the constituent SPP and ENZ modes. With  and  being the dispersion relations of the two oscillators in the complex angular frequency ω̃ and real transverse wavevector *k*_*x*_ space, we write the interaction Hamiltonian of the coupled system in the rotating wave approximation as^39,40^
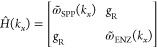
1where *g*_R_ is the coupling strength, also known as the vacuum Rabi splitting. The complex frequency , where *l* = {SPP, ENZ}, ω_*l*_ is the resonance frequency of mode *l* at wavevector *k*_*x*_ and γ_*l*_(*k*_*x*_) is the associated damping. The eigenfrequencies  of  (also known as the Hopfield or Hopfield–Bogliubov matrix^34,39^) form the dispersion relations of the hybrid modes and are given by
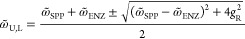
2where the suffixes U and L denote the upper and lower polaritons, respectively. The eigenvectors of  are also know as Hopfield coefficients, and their squared modulus denotes the relative mode fractions of the constituent SPP and ENZ modes in the hybrid polaritons at each *k*_*x*_.

**Figure 3:**
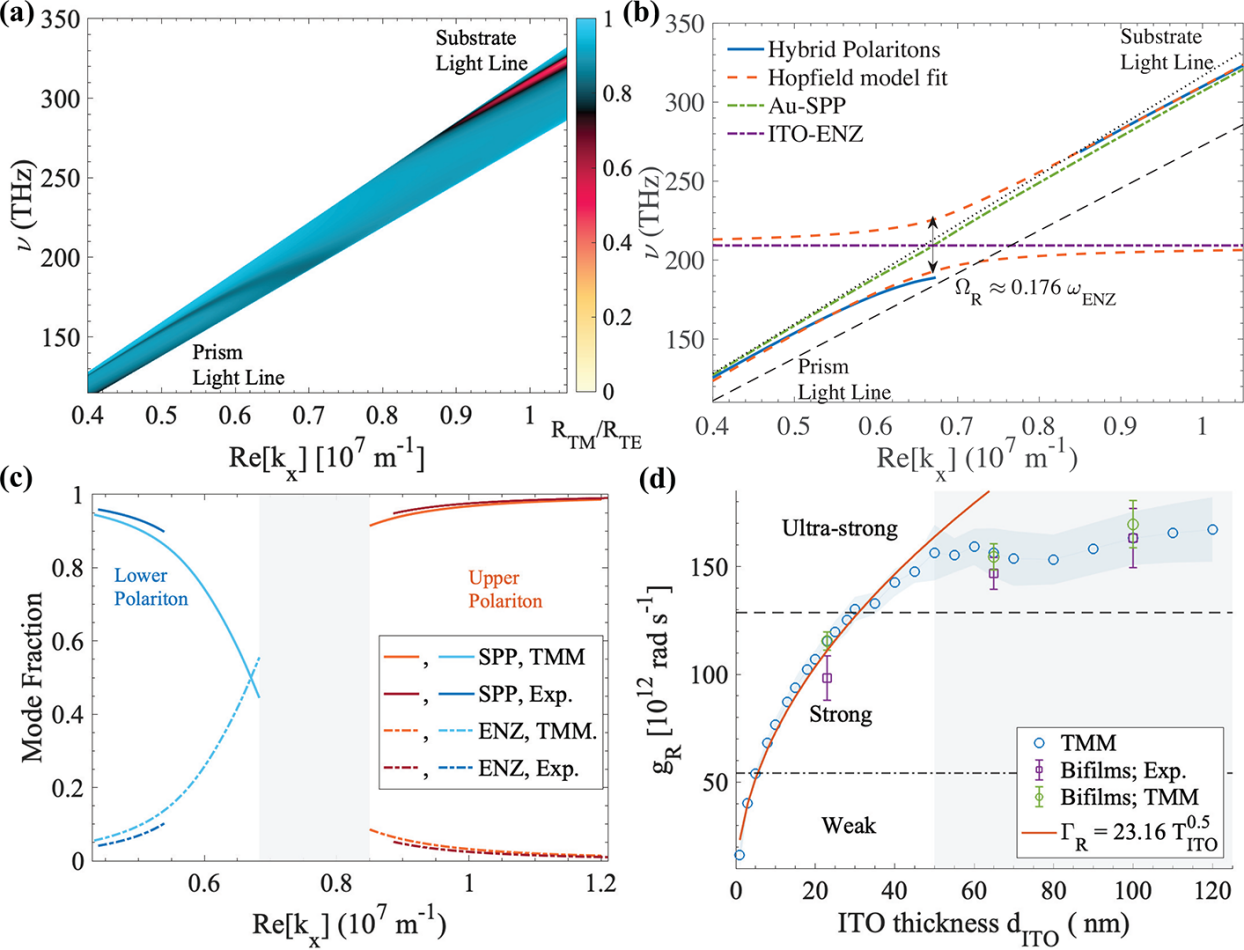
(a) Simulated reflectance map of bifilm A in *k*_*x*_–ν space. (b) Dispersion lines of the SPP mode (green, dot-dashed), the ENZ mode (purple, dot-dashed), the hybrid polaritons in bifilm A (blue, solid), and their Hopfield model fits (red, dashed). (c) The SPP (solid) and ENZ (dot-dashed) mode fractions for the upper (red and maroon) and lower (cyan and blue) polaritons. The upper (lower) polariton is formed by a symmetric (antisymmetric) superposition of the constituent modes. (d) *g*_R_ for bifilms with various values of *d*_ITO_ estimated from the simulated (green circles) and measured (purple squares) reflectance maps of bifilms A, B, and C and the simulated reflectance maps of bifilms with ϵ_ITO_ assumed to be the same as in bifilm A (blue circles). The error bars for estimated *g*_R_ from simulations for *d*_ITO_ < 80 nm and from measurements for bifilm A are given by the difference in *g*_R_ from fitting the upper and the lower polariton dispersion lines. The 95% confidence intervals for *g*_R_ estimated from fitting only the upper polariton dispersion line form the error bars for estimated *g*_R_ from simulations for *d*_ITO_ ≥ 80 nm and from measurements for bifilms B and C. The red line is the parabolic fit to *g*_R_ outside the gray region in which the Hopfield model yields large fitting errors.

We calculate the dispersion lines  and  from their respective reflectance maps in the un-normalized wavevector (*k*_*x*_) and frequency (ν) space by fitting an “asymmetric” Lorentzian with a frequency-dependent line width (eqs 1 and 2 in the Supporting Information) to the reflectance spectrum at each *k*_*x*_. The prism dispersion reshapes the reflectance maps in the *k*_*x*_/*k*_0_–ν space (Figure 2(a) for bifilm A, for example), so as to confine them between the light lines of the prism and the substrate (Figure 3(a) for bifilm A) in the *k*_*x*_–ν space. The polaritons thus have a positive group velocity.

For the ENZ mode, we assume a flat dispersion line^18^ as follows:
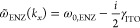
3where ω_0,ENZ_ is the ENZ mode frequency and γ_ITO_ is the damping in the Drude model of the permittivity of ITO, which is written as
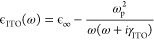
4Here, ϵ_*∞*_ is the asymptotic value of permittivity for frequencies much larger than the ENZ frequency, ω_p_ is the plasma frequency, and we have neglected nonlocal contributions to ϵ_ITO_(ω).^41^ For the ITO in bifilm A, ϵ_*∞*_ = 3.901, ω_p_ = 2.8533 × 10^15^ rad s^–1^, and γ_ITO_ = 2.116 × 10^14^ rad s^–1^. For the ITO in bifilm B, ϵ_*∞*_ = 3.6914, ω_p_ = 2.6667 × 10^15^ rad s^–1^, and γ_ITO_ = 1.193 × 10^14^ rad s^–1^. And for the ITO in bifilm C, ϵ_*∞*_ = 3.7359, ω_p_ = 2.6948 × 10^15^ rad s^–1^, and γ_ITO_ = 1.289 × 10^14^ rad s^–1^. We calculate *g*_R_ by performing a nonlinear least-squares fit of the hybrid polariton dispersion lines to their Hopfield model expressions in eq 2. Since our assumption of a flat  pinned at the ENZ frequency of ITO is not accurate for all *d*_ITO_ considered here, we take ω_0,ENZ_ to be an adjustable parameter and minimize the difference in *g*_R_ obtained from fitting the upper and the lower polariton. See Section S4 in the Supporting Information for details.

Figure 3(b) shows the simulated dispersion lines of the hybrid polaritons of bifilm A (blue, solid), their Hopfield model fits (red, dashed), and the dispersion lines of SPP (green, dot-dashed) and ENZ modes (purple, dot-dashed). The Hopfield fits plotted for all *k*_*x*_ agree reasonably well with the simulated bifilm dispersion, and they clearly show the avoided crossing. The estimated value of *g*_R_ for bifilm A from the fits is 115.5 ± 4.3 × 10^12^ rad s^–1^ and, being significantly larger than the average decay rate of the constituent modes γ_avg_ [= (γ_SPP_ + γ_ENZ_)/2 ≈ 54 × 10^12^ rad s^–1^], clearly satisfies the strong coupling criterion. The polariton band gap Ω_R_ (= 2*g*_R_) is approximately 0.176ω_0,ENZ_, which is close to the ultrastrong coupling threshold where Ω_R_ ≥ 0.2ω_0,ENZ_.^36,37^ Above this threshold, depolarization effects within the ITO film and the related counter-rotating terms, which are not included in our calculation of *g*_R_, become significant.^42^

From the Hopfield fit to the upper polariton mode, we note that its avoided crossing is pushed to the left of the substrate light line due to the dispersion of the coupling prism and is therefore not accessible. Hence, only its mostly SPP-like tail lies between the two light lines. The predominantly SPP-like nature of the upper polariton is also evident in its mode composition, which, as shown in Figure 3(c), has an SPP fraction larger than 0.9 throughout. We also note from Figure 3(c) that the ENZ mode contribution for both hybrid polaritons increases closer to the avoided crossing. Comparing the simulated dispersion line of the lower polariton and its Hopfield fit in Figure 3(b), we note that its dispersion line is not well-defined for wavevectors beyond the avoided crossing as a consequence of its largely ENZ-like nature at those wavevectors. Here, both the spectral line width and the wavevector uncertainty of the reflectance dip broaden as the absorption losses increase and the dispersion flattens.^2,3^

Since the spatial overlap between SPP and ENZ modes in the bifilm determines *g*_R_ (and Ω_R_), it can be varied using the material and geometrical parameters of the ITO film. The SPP mode is confined to the gold–ITO interface with a long evanescent tail extending into the substrate, while the ENZ mode is mostly constant and localized to the ITO film. Hence, as we observe in Figure 2, Ω_R_ initially increases with *d*_ITO_. Figure 3(d) shows the estimated *g*_R_ for various values of *d*_ITO_. For *d*_ITO_ smaller than 7 nm, *g*_R_ increases almost linearly but remains below the strong coupling threshold. Above this threshold, *g*_R_ is proportional to , as shown by the fitted curve (red, solid), and exceeds the ultrastrong coupling threshold for *d*_ITO_ larger than 30 nm. The major factor determining this scaling is that the ENZ mode becomes more LR-SPP-like as *d*_ITO_ increases, and the *E*_*z*_ field within the ITO film is no longer constant.^18^ Furthermore, the ENZ mode is a collective excitation of the free electrons within the ITO film with an oscillator strength *f*_ENZ_ that scales with *d*_ITO_. Since *g*_R_ is proportional to ,^2,34^*g*_R_ should scale with . For *d*_ITO_ larger than 45 nm, *g*_R_ saturates and deviates from the  dependence as the ENZ mode transforms into an LR-SPP mode at these thicknesses, and its dispersion can no longer be approximated by a flat line given by eq 3. We have identified this range of *d*_ITO_ by a shaded gray region in Figure 3(d), and the values of *g*_R_ extracted from the analytical model in this region are not accurate, which is also reflected in the large fitting errors (blue shaded area) in this region. For *d*_ITO_ larger than 65 nm, the fields at the two interfaces of the ITO film also start to decouple, and the hybrid polaritons morph into polaritonic modes confined at these interfaces.^34^ Thus, *d*_ITO_ ≤ 45 nm provides an upper limit to *g*_R_ that can be achieved with modes that inherit the desirable features of both the ENZ mode and the SPP mode. See Sections S5 and S7 in the Supporting Information for further discussion.

**Figure 4:**
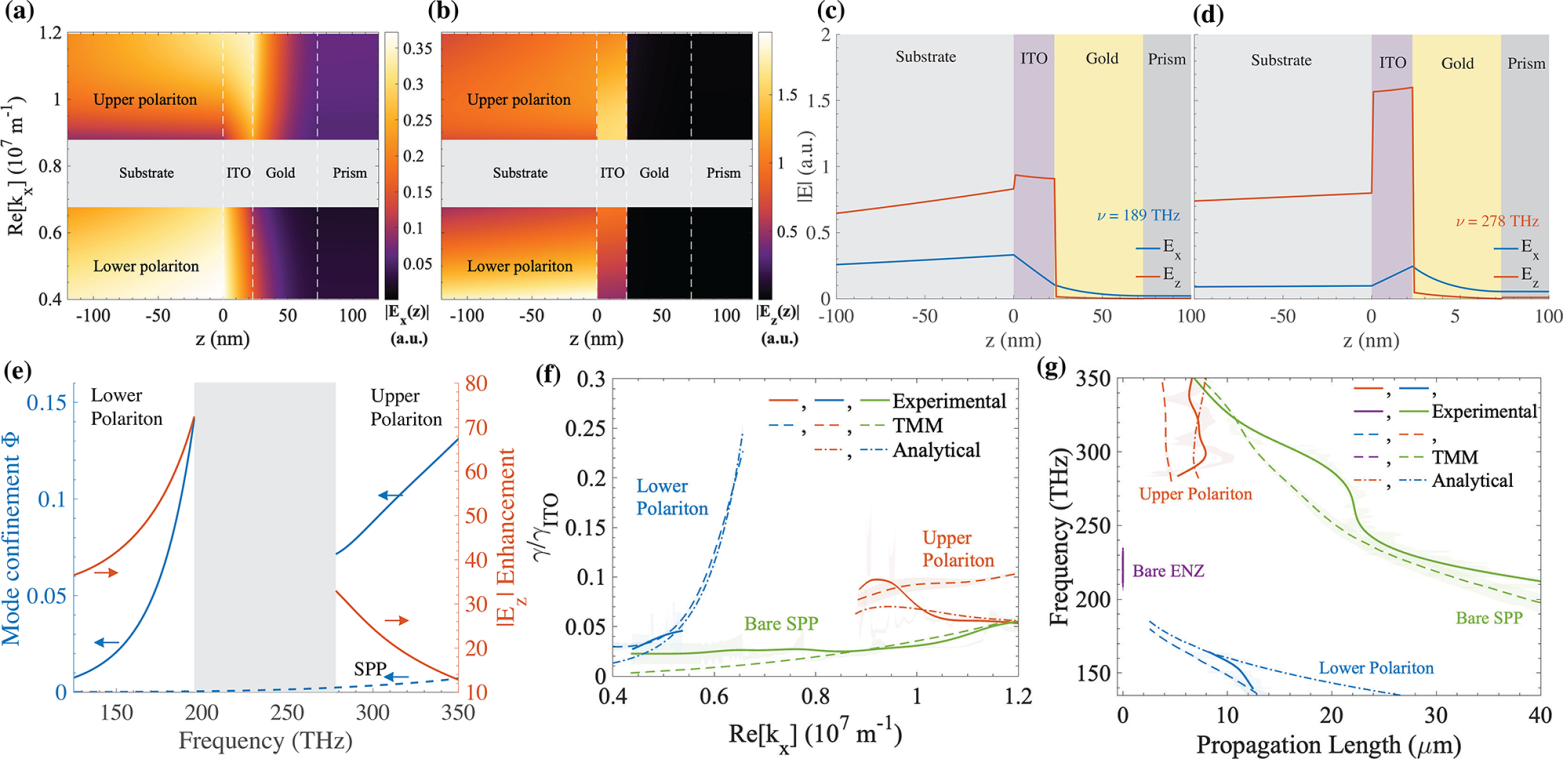
Profiles of (a) |*E*_*x*_| and (b) |*E*_*z*_| for bifilm A plotted along the dispersion lines of the hybrid polaritons. The polariton band gap is shown in gray, and the interfaces by the white dashed lines. Field distributions of |*E*_*x*_| (blue) and |*E*_*z*_| (red) for the (c) lower and the (d) upper polariton at the edges of the band gap. (e) Mode confinement of the same hybrid polaritons (blue, solid) and the SPP mode (blue, dashed) and their longitudinal field enhancement in ITO with respect to the field in gold at the gold–ITO interface (red). (f) Damping γ normalized to the decay constant in the Drude model of ITO γ_ITO_ and the (g) propagation lengths of the upper (red) and lower (blue) polaritons of bifilm A, the SPP mode in an isolated 50-nm-thick gold film (green), and the ENZ mode in an isolated 23-nm-thick ITO film (purple). In (f) and (g), the dot-dashed lines are the solutions of the analytical dispersion relation, and the solid (experiment) and the dashed (TMM simulations) lines are the line widths of the dips in the respective reflectance maps smoothed over their fitting errors (shaded areas around the lines).

The relevance of these hybrid polaritons to photonic applications can be examined through parameters such as their mode confinement, field enhancement, propagation lengths, and decay rates. Following the method described in appendix B of ref (43) we first develop an analytical dispersion model for the polaritons and then use its solutions to calculate their field profiles, mode confinement, and field enhancement. See Sections S6 and S7 in the Supporting Information for details on these calculations. We restrict our discussion from now on to bifilm A. Section S9 in the Supporting Information has details on the polaritons in bifilms B and C. Figure 4(a) and (b) show the transverse |*E*_*x*_| and the longitudinal |*E*_*z*_| electric field profiles, respectively, of the polaritons in bifilm A plotted at various *k*_*x*_ along their dispersion lines. From the continuity of the longitudinal component of the electric flux density *D*_*z*_ at the gold–ITO interface, we have *E*_z,ITO_ = (ϵ_Au_/ϵ_ITO_)*E*_z,Au_, where *E*_z,ITO_ (*E*_z,Au_) is the longitudinal electric field inside ITO (gold) at the interface and ϵ_ITO_ (ϵ_Au_) is its permittivity. As ϵ_ITO_ vanishes close to the avoided crossing, *E*_*z*_ is significantly enhanced within the ITO film and relayed from the gold–ITO interface to the substrate while maintaining its large amplitude.^18,44,45^ Away from the avoided crossing, both polaritons become more SPP-like with a smaller *E*_*z*_ in ITO and *E*_*x*_ confined along the ITO–substrate (gold–ITO) interface for the lower (upper) polariton. Figure 4(c) and (d) show the mode profiles of |*E*_*x*_| (blue) and |*E*_*z*_| (red) of the upper and the lower polariton, respectively, at the edges of the band gap (the gray region in Figure 4(a) and (b)). The hybrid nature of the modes is evident in the large amplitude of |*E*_*z*_| within ITO and an enhanced |*E*_*x*_| at the edges of the ITO film.

Figure 4(e) shows the enhancement in *E*_*z*_ for the hybrid polaritons in bifilm A, which is defined as |*E*_*z*_| at the center of the ITO film normalized to the |*E*_*z*_| in gold near the gold–ITO interface (red, solid). We note that the lower (upper) polariton can have a field enhancement as large as 75× (32×) close to the avoided crossing. We now define the mode confinement Φ as follows:^46^
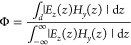
5where^43^*H*_*y*_(*z*) = (*ωϵ*(*z*))/(μ_0_*ck*_*x*_)*E*_*z*_(*z*) is the magnetic field and *d* denotes the integration range of *z*. For the bifilm, we calculate Φ only within the ITO layer, whereas we calculate Φ within the gold layer for the standalone SPP mode and within the ITO layer for the standalone ENZ mode. Figure 4(e) shows the variation of Φ for the polaritons in bifilm A (blue, solid) and the SPP mode in the standalone gold film (blue, dashed). We observe that although Φ for both hybrid polaritons is lower than the bare ENZ mode (≈0.3, not shown here), they substantially outperform the bare SPP mode throughout the spectral region of interest with values of Φ approaching 0.14 (0.075), close to avoided crossing for the lower (upper) polariton. This relaxation in mode confinement makes the hybrid polaritons less lossy compared to the ENZ mode, which is reflected in their reduced damping and enhanced propagation lengths.

Figure 4(f) shows the damping , and Figure 4(g) the propagation lengths (= 1/(2Im[*k*_*x*_])) of the upper (red) and the lower (blue) polaritons of bifilm A, the SPP mode in the standalone gold film (green), and the ENZ mode in the standalone ITO film (purple). Asymmetric Lorentzians fitted to the spectral dips at each Re[*k*_*x*_] in the experimental (solid) and the simulated (dashed) reflectance maps yield γ, while the fits to the wavevector scans at each frequency yield Im[*k*_*x*_]. See Sections S5 and S8 in the Supporting Information for details on these fits. The values of γ and Im[*k*_*x*_] in the analytical dispersion model (dot-dashed) are given by the imaginary part of the complex frequency and the complex wavevector solutions to the dispersion relation for each Re[*k*_*x*_] and , respectively. The estimated damping and propagation lengths in both the simulated and the experimental data sets have a reasonable agreement in the presence of fitting errors and differences between the simulated and experimental optical constants. The results from the analytical model exclude the radiative losses into the coupling prism.^13^ The hybrid polaritons have a significantly lower damping throughout compared to the ENZ mode, which has a constant damping of 0.5γ_ITO_ (not shown in Figure 4(f)).^18^ Additionally, the lower (upper) polariton has a propagation length between 2 and 15 μm (4–8 μm) for bifilm A, which is significantly larger than the propagation length of the ENZ mode (≈ 0.08 μm for the 23-nm-thick ITO film). The damping (propagation length) of the lower polariton is maximized (minimized) close to the avoided crossing and approaches the values for the SPP mode away from it.

## Conclusion

To summarize, we have proposed a bifilm structure consisting of a 50-nm-thick gold film deposited on a thin ITO film backed by a float glass substrate that supports hybrid polaritons formed by strong coupling between the SPP mode in the gold film and the ENZ mode in the ITO film at NIR frequencies. These polaritons have a much tighter mode confinement than the bare SPP mode, along with a propagation length of several microns in contrast to the nonpropagating ENZ mode. The large mode confinement of these polaritons is accompanied by a significant enhancement in the longitudinal component of the electric field within the ITO film. The coupling between the constituent modes can be tuned through the thickness of the ITO layer and can even approach the ultrastrong coupling regime at certain thicknesses.

A propagation length of several microns implies an interaction length of several wavelengths at these NIR frequencies. This large interaction length along with the tight mode confinement and the large sub-picosecond nonlinear response of ITO in its ENZ region^16^ make our device an ideal platform for electro-optical control of strong coupling,^47^ ultrafast switching,^48^ and studying giant ultrafast nonlinearities that do not rely on lossy optical resonances or require sophisticated fabrication techniques. The use of a prism for coupling to the polaritons can also be done away with through the use of appropriate grating couplers.^49^ The ultrafast response of ITO should also allow for the observation of effects due to time refraction and adiabatic frequency conversion^50−52^ and exotic effects related to ultrastrong coupling phenomena, such as the dynamic Casimir effect.^53^

## Methods

### Sample Fabrication

The bifilm samples were fabricated by depositing a 50-nm-thick layer of gold on commercially available ITO films on a float glass substrate through thermal evaporation. First, the ITO films were cleaned through the use of acetone + extreme sonication followed by IPA + extreme sonication to remove most of the contamination over the surface that could lead to the undesired scattering of the surface waves. A thermal source was then used to evaporate the gold at a constant rate until a 50 nm layer of Au accumulated over the samples under a high vacuum. No adhesion layer was used between the gold layer and the substrate. Attenuated total reflection spectroscopy in a Kretschmann configuration was used to characterize the dispersion of the fabricated samples by measuring their reflectance maps. The schematic of the setup used for measurements is shown in Figure S2 in Section S2 of the Supporting Information along with the measurement details.

### Simulations and Analytical Modeling

The TMM simulations were performed using our home-built MATLAB code. The dispersion of gold, ITO, float glass, and prism were included by using their respective frequency-dependent permittivities. We used the data from Johnson and Christy for the permittivity of gold.^38^ The permittivity spectra of the ITO samples used in the three bifilms are shown in Figure S1(a)–(c) in the Supporting Information. The permittivity of Schott N-BK7 was used for the float glass substrate.

The dispersion of the hybrid polaritons in the bifilms was analytically modeled through use of the method described in Appendix B of ref (43). The electric field in each layer was given by a coherent sum of evanescent wave-like forward and backward propagating guided solutions. The field continuity relations at each interface of the structure yielded a set of eight linear homogeneous equations for the field coefficients in each layer, which could be written in the matrix form. The analytical dispersion was obtained by minimizing the determinant of this coefficient matrix in either the complex frequency and real wavevector space or the real frequency and complex wavevector space using the Nelder–Mead method. The field coefficients were obtained through singular value decomposition of the coefficient matrix at each solution in the complex frequency and real wavevector space obtained from the analytical dispersion model. The mode profiles were then calculated by substituting the field coefficients in the aforementioned expressions for the electric field distributions in each layer. See Sections S6 and S7 in the Supporting Information for more details on the analytical model.

## Supporting Information Available

Permittivity spectra of ITO used in the bifilm samples; setup diagram and measurement details; simulated and measured reflectance maps of standalone gold and standalone ITO films; discussion on the relatively smaller visibility of the lower polariton branch; description of the procedure to estimate the coupling strength *g*_R_; details of the analytical dispersion model used to calculate the field profiles of the polaritons; description of the procedure to calculate the field profiles and discussion of the field profiles of bifilms B and C; description of the model used to estimate propagation lengths of the polaritons from their reflectance maps (PDF)
