# Supplementary material for: Strongly Coupled Plasmon Polaritons in Gold and Epsilon-Near-Zero Bifilms
Source: ACS Photonics. 2023 Jan 3;10(1):162–9. doi: 10.1021/acsphotonics.2c01412 (PMC9853859; doi:10.1021/acsphotonics.2c01412)
Supplement: Supplementary file 1 — ph2c01412_si_001.pdf [file ph2c01412_si_001.pdf]

Supplementary Information for "Strongly Coupled Plasmon Polaritons in Gold and Epsilon-Near-Zero Bifilms"

Saumya Choudhary,[*,†] Saleem Iqbal,[†] Mohammad Karimi,[‡] Orad Reshef,[‡] M.Zahirul Alam,[‡] and Robert W. Boyd[‡,†]

†*Institute of Optics, University of Rochester, Rochester NY 14627, USA*

‡*Department of Physics, University of Ottawa, Ottawa, ON K1N 6N5, Canada*

E-mail: schoudha@ur.rochester.edu

- Number of pages: 21
- Number of figures: 11
- Number of tables: 0

S1. Permittivity of indium tin oxide used in the bifilm samples

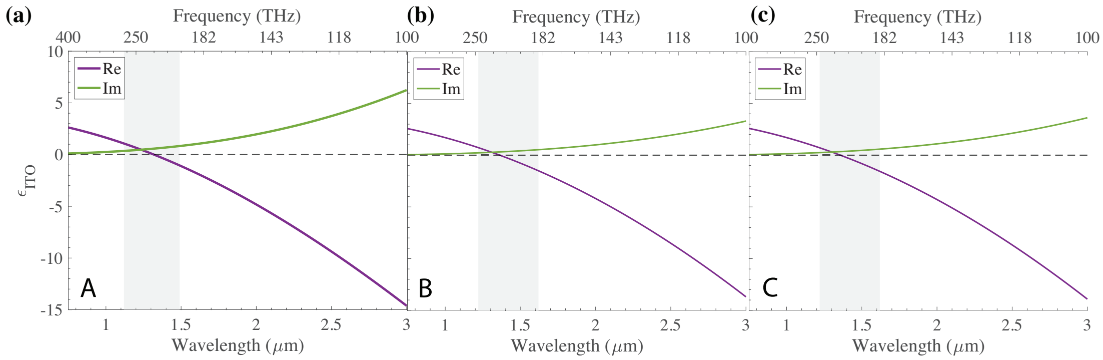

Figure S1: Spectra of the real (purple) and the imaginary (green) parts of the permittivity of ITO in bifilm samples (a) A, (b) B and (c) C, respectively. The gray region denotes the epsilon-near-zero (ENZ) spectral region.

Figure S1 shows the spectra of the real (purple) and the imaginary (green) parts of the permittivies of the ITO samples used in bifilms A (a), B (b) and C (c), respectively. The Re[ϵ] approaches zero at a frequency (wavelength) of 227.79 THz (1.317 μm) for sample A, 220.08 THz (1.363 μm) for sample B, and 220.94 THz (1.357 μm) for sample C.

S2. The experimental setup

Figure S2 shows the schematic of the experimental setup used to measure the reflectance maps through attenuated total reflection spectroscopy in a Kretschmann configuration. Broadband light from the halogen lamp is collimated using the objective O1. The exit pupil of the objective is imaged on the bifilm sample (cross-section shown in the inset) using the lens L1. The aperture A adjusts the spot size to avoid clipping the beam from the edges of the prisms. The thin film polarizer P transmits the TM-polarized component. The bifilm sample is kept in contact with an N-SF11 prism Pr1 with index-matching oil in between (Cargille series M 1.780). Another N-SF11 prism Pr2 redirects the reflected light from the diagonal face of the prism (and the sample) parallel to the incoming light. The prism and

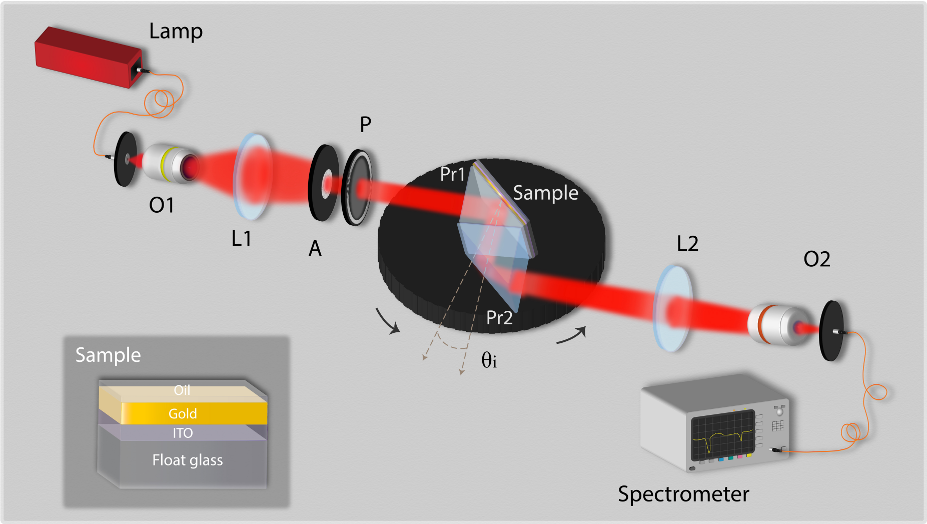

Figure S2: Schematic of the experimental setup for measuring the reflectance maps using attenuated total reflection (ATR) spectroscopy in the Kretschmann-Raether[1] configuration. The cross-section of the sample is shown in the inset.

sample assembly is mounted on a rotation mount to vary the incidence angle on the sample. Another lens L2 images the sample onto the entrance pupil of a microscope objective O2, which couples the reflected light to a multi-mode fiber coupled spectrometer. We use an optical spectrum analyzer OSA (Agilent 86142A) to record the spectra from 600 nm to 1600 nm. We use an InGaAs spectrometer (customized SM304 from Spectral Products) to record the spectra from 1600 nm to 2300 nm.

We take into account the shape of the blackbody spectrum of the lamp, the spectral response of the prisms and the oil, and the optical elements used in the setup by normalizing the TM-polarized reflectance spectrum from the sample at a particular incidence angle to the TE-polarized reflectance spectrum at the same angle. Before the spectral normalization, the detector noise in each spectrum is smoothed over first by using a Savtizky-Golay filter, and then by applying the wavelet transform.

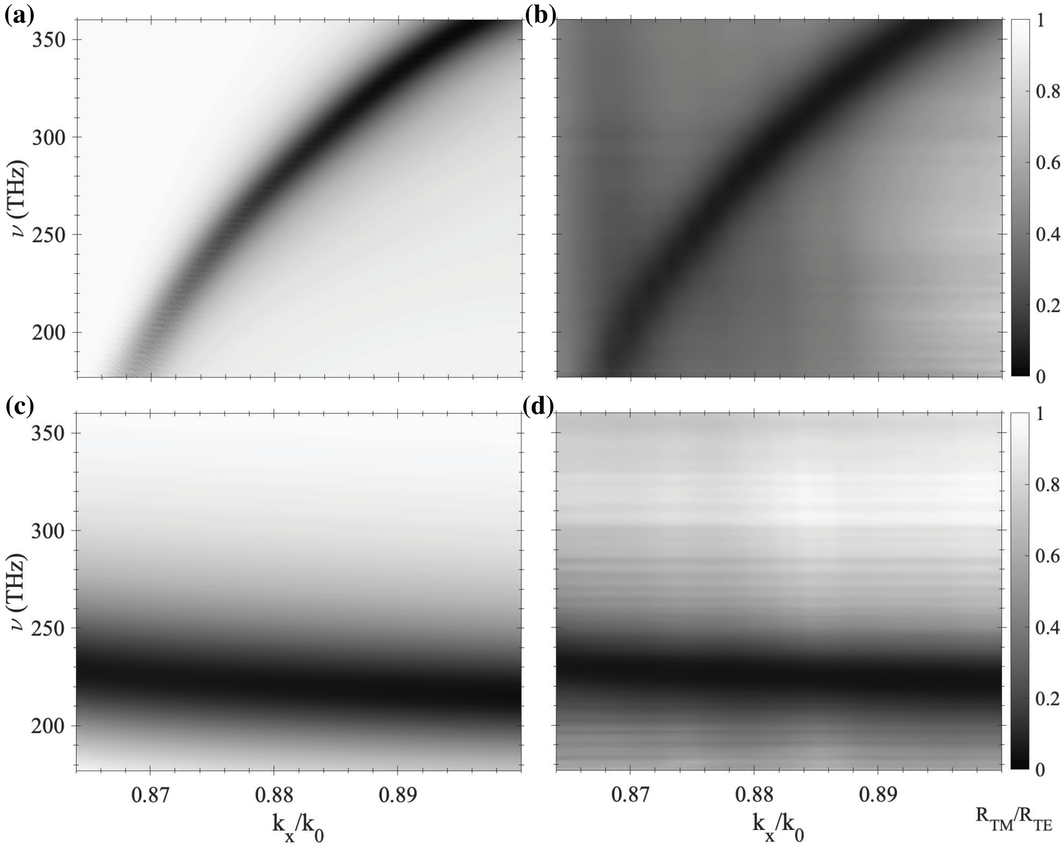

Figure S3: Reflectance maps of the standalone gold sample (top panels), and the standalone 23 nm thick ITO sample (bottom panels) obtained from TMM simulations (left), and measured experimentally (right).

S3. Reflectance maps of the standalone constituent modes

The reflectance (R_{TM}/R_{TE}) maps of the standalone gold sample are shown in the top panels in Figure S3, while the reflectance maps of the standalone 23 nm thick ITO (permittivity spectrum shown in fig S1(a)) sample are shown in the bottom panels. The simulated (measured) results are shown on the left (right) panel. The frequencies of the reflectance dips of the SPP mode in the standalone gold sample are strongly wavevector dependent, while the frequencies of the reflectance dips of the ENZ mode in the standalone ITO sample stay almost constant for all wavevectors.

S4. The visibility of the lower polariton branch

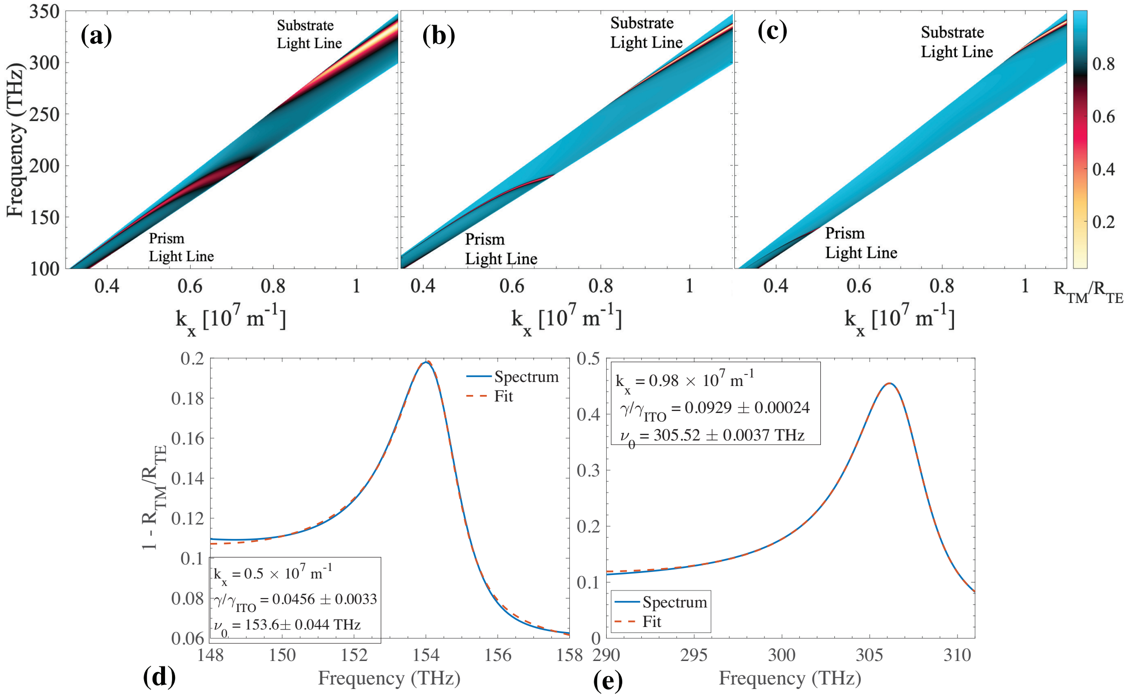

Figure S4: Reflectance map R_{TM}/R_{TE} obtained from TMM simulations in the un-normalized wavevector and frequency space of (a) a bifilm with 35-nm-thick gold and 23-nm-thick ITO with the same permittivity as in bifilm A, and of (b) bifilm A, and (c) bifilm C with reduced losses in the ITO film. The spectra of ϵ_{ITO} used in (b) and (c) are shown in Figures S1(a) and S1(c), respectively but with $\text{Im}[\epsilon_{ITO}]$ reduced by a factor of 10. Asymmetric lorentzian fits (red, dashed) to the extinction spectrum ($(1 - R_{TM}/R_{TE})$, blue, solid) of bifilm A for the (c) lower, and the (d) upper polariton at a certain $\text{Re}[k_x]$ (shown in the inset of each plot). The 95% confidence intervals of the fit parameters $[\omega_0/(2\pi, \gamma)]$ are stated in the inset.

The critical coupling condition for our bifilm structure at each frequency ν, or when the coupling losses are balanced by the absorption losses,[1] are given by the solutions to the characteristic equation in the complex wavevector k_x and real frequency ν space for which the $\text{Im}[k_x]$ is minimized. These solutions depend on the geometrical parameters of the bifilm. The same set of parameters do not satisfy the critical coupling criterion for both the upper and the lower polariton simultaneously due to large polariton band gap. Figure S4(a) shows the reflectance map of a bifilm with a 35-nm-thick gold film and 23-nm-thick ITO film obtained from TMM simulations in the frequency ν and un-normalized wavevector k_x space. The permittivity of the ITO layer is taken to be the same as bifilm A. Comparing

Figure S4(a) with the reflectance map of bifilm A shown in Figure 3(a) of the main text, we note that the choice of a thinner gold film in the former case leads to a better coupling efficiency to the lower polariton. However, the thinner gold film also leads to larger radiative damping of the upper polariton, which is evident in its broader spectral linewidth. Hence, the choice of a 50-nm-thick gold layer for our bifilms leads to efficient (inefficient) coupling to the upper (lower) polariton.

Large absorption losses within the ITO film also contribute to the smaller amplitude of the resonance dip for the lower polariton compared to the upper polariton. Figure S4(b) shows the reflectance map of bifilm A obtained from TMM simulations in the frequency ν and un-normalized wavevector k_x space, wherein the losses in the ITO film have been reduced by substituting the ITO permittivity ϵ_{ITO} to be $(\mathrm{Re}[\epsilon_{\mathrm{ITO}}] + i\mathrm{Im}[\epsilon_{\mathrm{ITO}}]/10)$ in the TMM calculation. The wavevector k_x is defined such that $\mathrm{Re}[k_x(\omega)] = (\omega/c)\sin[\pi/4 + \arcsin(\sin\theta_i/n_p(\omega))]$, where $n_p(\omega)$ is the refractive index of the prism, and θ_i is the rotation angle of the prism-sample system (shown in Figure S2). The reduced losses in the ITO film lead to much sharper dips for both polariton branches when compared with the reflectance map calculated with the full permittivity of the ITO film ϵ_{ITO} (Figure 3(a) in the main text). Additionally, the reduced losses in ITO result in a well-defined lower polariton branch that continues to exist until it approaches the prism light line. Figure S4(c) shows the reflectance map of bifilm C with similarly reduced losses in the ITO layer, and we see a well defined lower polariton branch that is pushed to smaller frequencies than for the thinner ITO in bifilm A. This lower polariton branch is not as clearly visible with the full ITO permittivity ϵ_{ITO}. Hence, the losses in the ITO layer are responsible for increasing both the wavevector uncertainty, and the spectral linewidth of the lower polariton branch close to the avoided crossing region where the ENZ mode fraction becomes increasingly significant. Both factors also contribute to the reduced visibility of the lower polariton branch in comparison with the upper polariton branch.

S5. Estimating the coupling strength g_R

We obtain the dispersion lines of the hybrid polaritons in the bifilm, and the SPP mode in the complex frequency $\tilde{\omega}\,(=\omega_0 - i\gamma)$, real wavevector $\mathrm{Re}[k_x]$ space by fitting an asymmetric Lorentzian $f_{\mathrm{Re}[k_x]}(\omega)$ defined below, to the extinction $(1 - R_{TM}/R_{TE})$ spectra at each $\mathrm{Re}[k_x]$[2]

$$f_{\mathrm{Re[k_x]}}(\omega) = \frac{2A/\pi\gamma(\omega)}{1 + [(\omega - \omega_0)/\gamma(\omega)]^2} + B + C\omega, \tag{1}$$

where

$$\gamma(\omega) = \frac{2\gamma_0}{1 + e^{a(\omega - \omega_0)}}. \tag{2}$$

Here A is a fitting parameter that determines the peak of the extinction spectrum, and a is the asymmetry parameter. When a is zero, we recover the normal symmetric Lorentzian function with a full width at half maximum linewidth of $2\gamma_0$. The fit parameters B and C account for the frequency dependent linear distortion of the extinction spectra due to the dispersion of the prism.

From the dispersion lines of the SPP mode $\tilde{\omega}_{\mathrm{SPP}}(k_x)$, and the upper and the lower hybrid polaritons $\tilde{\omega}_{\mathrm{U,L}}(k_x)$ calculated from their respective reflectance maps, we estimate the coupling strength g_R of the bifilm system by fitting the eigenvalues of the Hopfield-Bogliubov interaction Hamiltonian matrix,[3] shown in equation (3), to the upper and the lower polariton dispersion lines

$$\tilde{\omega}_{\mathrm{U,L}} = \frac{\tilde{\omega}_{\mathrm{SPP}} + \tilde{\omega}_{\mathrm{ENZ}} \pm \sqrt{(\tilde{\omega}_{\mathrm{SPP}} - \tilde{\omega}_{\mathrm{ENZ}})^2 + 4g_R^2}}{2}. \tag{3}$$

We assume that the ENZ mode has a flat dispersion line given by

$$\tilde{\omega}_{\mathrm{ENZ}}(k_x) = \omega_{0,\mathrm{ENZ}} - i\gamma_{\mathrm{ITO}}/2, \tag{4}$$

where γ_{ITO} is the damping in the Drude permittivity model of ITO, and the resonance

frequency $\omega_{0,\text{ENZ}}$ is close to the ENZ frequency of ITO given by

$$\omega_{\text{ENZ,ITO}} = \sqrt{\frac{\omega_P^2}{\epsilon_\infty} - \gamma_{\text{ITO}}^2}, \quad (5)$$

where ω_P is the plasma frequency, and ϵ_∞ is the asymptotic value of permittivity for frequencies much larger than the ENZ frequency in the Drude permittivity model of ITO. We take $\omega_{0,\text{ENZ}}$ to be an adjustable parameter in our fit, and not equal to $\omega_{\text{ENZ,ITO}}$ as this assumption only holds true for an infinitesimally small thickness of the ITO film.[4] As the ITO thickness increases, the symmetric polariton mode within the ITO film transitions from an ENZ mode, which is asymptotically pinned to $\omega_{\text{ENZ,ITO}}$, to a long-range surface plasmon polariton (LR-SPP) mode, whose dispersion line asymptotically approaches lower frequencies than $\omega_{\text{ENZ,ITO}}$ for large wavevectors.

We begin with $\omega_{0,\text{ENZ}} = \omega_{\text{ENZ,ITO}}$, and use the nonlinear least squares method to individually fit the dispersion lines of the upper and the lower polaritons to their analytical dispersion relations given in equation (3). If the difference between the two values of g_R that we obtain from the upper and the lower polariton fits is larger than 10%, we repeat the curve fit albeit with a slightly reduced value of $\omega_{0,\text{ENZ}}$ until the difference in g_R obtained from both the upper and the lower polariton fits is minimized. The average of the two g_R values obtained after this optimization procedure is the final calculated value of the coupling strength of the hybrid polaritons, and the values themselves form the confidence interval of the fit.

We note that as the thickness of the ITO film d_{ITO} is increased, the assumption of a flat dispersion line for the ENZ mode at all wavevectors no longer holds true as the mode becomes more LR-SPP like in nature, even with the optimization of its resonance frequency. This effect manifests in relatively larger confidence intervals of g_R in the fit (shown by the shaded blue region in Figure 3(d) of the main text) for $d_{\text{ITO}} > 65$ nm than for bifilms with thinner ITO. Additionally, g_R itself also increases as d_{ITO} is increased due to an enhancement

of the oscillator strength of the ENZ mode. Consequently, as discussed in the main text, g_R becomes larger than $0.1\omega_{0,\mathrm{ENZ}}$, where $\omega_{0,\mathrm{ENZ}}$ is the avoided crossing frequency, and the SPP and the ENZ modes in the bifilm are ultra-strongly coupled for d_{ITO} larger than 30 nm.

In this ultra-strong coupling regime, the simple analytical (Hopfield) model for the hybrid polaritons based on two coupled harmonic oscillators is not accurate due to several underlying assumptions made by the model. For instance, the self-interaction term of the SPP and the ENZ modes, which is proportional to the square of their respective polarizations, is neglected in the interaction Hamiltonian.[5] In the complete interaction Hamiltonian written in the dipole gauge, this self-interaction term is responsible for the renormalization of the respective uncoupled mode frequencies. In the quantum-mechanical picture, this term corresponds to the $\mathbf{A}^2$ term of the interaction Hamiltonian, which also includes the counter-rotating terms that are neglected when making the rotating-wave approximation (RWA). This self-interaction term leads to deviations from the curves obtained from the Hopfield model.

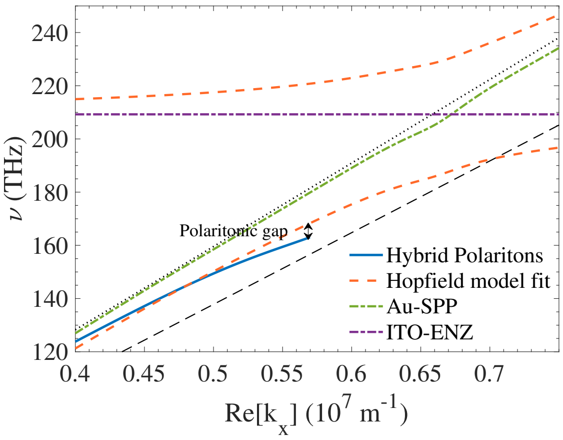

Figure S5: Dispersion lines of the hybrid polaritons for a bifilm with a 50 nm thick gold on a 40 nm thick ITO film (blue, solid), its Hopfield model fit (red, dashed), the SPP mode in the standalone 50 nm thick gold film (green, dot-dashed), and the ENZ mode in the 40 nm thick ITO film with permittivity shown in Figure S1(a). The black dashed and dotted lines are the prism and the substrate light lines, respectively.

For intersubband polaritons formed by ultra-strong coupling between a microcavity mode and the bound states in multiple quantum wells (which form a collective Berreman mode for

a large enough number of quantum wells), it has been previously demonstrated that the low-energy polariton branch asymptotically approaches a smaller energy than the high-energy polariton close to the avoided-crossing.[6] As a consequence, the high reflectivity between the two energy asymptotes opens up a so-called "polaritonic gap" in the dispersion line of the intersubband polaritons. We observe a similar effect in the dispersion lines of our bifilms that support ultra-strongly coupled SPP and ENZ modes, that is for bifilms with $d_{\mathrm{ITO}} > 30$ nm. As shown in Figure S5, the frequency asymptote of the lower polariton (blue, solid) is smaller than the frequency at that specific transverse wavevector in the fitted dispersion line (red, dashed). The reflectance in this range of frequencies between the two frequency asymptotes, or in other words the polaritonic gap, is high. This polaritonic gap also implies a progressively worse agreement between the analytical dispersion model, and the actual dispersion of the hybrid polaritons for larger d_{ITO}. Hence, there is a larger difference in the values of g_R estimated from the upper, and from the lower polariton fit (shown by the blue shaded region in Figure3(d) in the main text) in the ultra-strong coupling region.

For d_{ITO} larger than 80 nm, as a consequence of the large losses within the ITO layer, and inefficient excitation in our Kretschmann configuration, the spectral dips corresponding to the lower polariton branch become faint enough that it is not possible to perform the asymmetric Lorentzian fits to extract the dispersion line $\tilde{\omega}_L(k_x)$. Hence, we only plot the g_R corresponding to the upper polariton fit in Figure 3(d) of the main text with $\omega_{0,\mathrm{ENZ}}$ taken to be the same as that obtained through optimization of the bifilm with d_{ITO} of 70 nm. The blue shaded region shows the 95% confidence interval of the fit parameter g_R. We also use just the upper polariton branch to estimate g_R for the measured reflectance spectra of bifilms B and C, as the lower polariton data is not available for these samples due to the limited spectral range of our source and spectrometers.

S6. The analytical dispersion model

We follow the method described in the appendix B of Ref.[7] to analytically model the dispersion relations of the hybrid polaritons from which we also calculate the field profiles, the mode confinement, field enhancement and the damping and propagation lengths in the absence of radiative losses. In our coordinate system, shown in the inset of Figure 1(c) of the main text, the polaritons propagate parallel to the interfaces of the layered films, which we assume to be aligned along the x-axis. The films themselves are arranged along the z-axis with the substrate-ITO interface located at $z = 0$, the ITO-gold interface at $z = d_{\mathrm{ITO}}$, and the gold-prism interface at $z = d_{\mathrm{ITO}} + 50$ nm.The TM-polarized guided solutions in each layer of this multi-layered plasmonic structure are evanescent wave-like, and can be written as

$$E_{xl} = e^{ik_x x}(a_l e^{k_{zl} z} + b_l e^{-k_{zl} z}), \tag{6}$$

$$E_{zl} = \Big(\frac{ik_x}{k_{zl}}\Big) e^{ik_x x}(-a_l e^{k_{zl} z} + b_l e^{-k_{zl} z}), \tag{7}$$

$$E_y = 0, \tag{8}$$

where $l = \{s, i, a, p\}$ is the index for the substrate, ITO, gold and prism layers, respectively, in this multi-layered structure; k_x is the transverse wavevector; a_l and b_l are the coefficients of the forward and backward propagating solutions within the layer that are determined by the field continuity relations, and k_{zl} is the longitudinal wavevector given by

$$k_{zl}^2 = k_x^2 - \Big(\frac{\omega}{c}\Big)^2 \epsilon_l, \tag{9}$$

with ϵ_l being the permittivity of the layer l. The continuity of E_{xl} and $D_{zl}(= \epsilon_l E_{zl})$ at each interface leads to a set of 8 linear homogeneous equations for the 8 field coefficients $C^T = \{a_s, b_s, a_i, b_i, a_a, b_a, a_p, b_p\}$ at each k_x. In matrix form, the set of equations can be

written as

$$LC = 0, \tag{10}$$

where L is given by

$$L = \begin{bmatrix} 0 & 1 & 0 & 0 & 0 & 0 & 0 & 0 \\ 1 & 1 & -1 & -1 & 0 & 0 & 0 & 0 \\ -\epsilon_p k_{za} & \epsilon_p k_{za} & \epsilon_a k_{zp} & -\epsilon_a k_{zp} & 0 & 0 & 0 & 0 \\ 0 & 0 & e^{k_{za} d_{\mathrm{Au}}} & e^{-k_{za} d_{\mathrm{Au}}} & -e^{k_{zi} d_{\mathrm{Au}}} & -e^{-k_{zi} d_{\mathrm{Au}}} & 0 & 0 \\ 0 & 0 & -\epsilon_a k_{zi} e^{k_{za} d_{\mathrm{Au}}} & \epsilon_a k_{zi} e^{-k_{za} d_{\mathrm{Au}}} & \epsilon_i k_{za} e^{k_{zi} d_{\mathrm{Au}}} & -\epsilon_i k_{za} e^{-k_{zi} d_{\mathrm{Au}}} & 0 & 0 \\ 0 & 0 & 0 & 0 & e^{k_{zi} d_{\mathrm{Au,ITO}}} & e^{-k_{zi} T_{\mathrm{Au,ITO}}} & -e^{k_{zs} d_{\mathrm{Au,ITO}}} & -e^{-k_{zs} d_{\mathrm{Au,ITO}}} \\ 0 & 0 & 0 & 0 & -\epsilon_i k_{zs} e^{k_{zi} d_{\mathrm{Au,ITO}}} & \epsilon_i k_{zs} e^{-k_{zi} d_{\mathrm{Au,ITO}}} & \epsilon_s k_{zi} e^{k_{zs} d_{\mathrm{Au,ITO}}} & -\epsilon_s k_{zi} e^{-k_{zs} d_{\mathrm{Au,ITO}}} \\ 0 & 0 & 0 & 0 & 0 & 0 & 1 & 0 \end{bmatrix}, \tag{11}$$

with $d_{\mathrm{Au,ITO}} = d_{\mathrm{Au}} + d_{\mathrm{ITO}}$, and d_{Au} is the thickness of the gold layer (fixed at 50 nm throughout). A non-trivial solution of the equation (10) exists only when $|\det(\mathrm{L})| = 0$, which gives the characteristic equation of the bifilm. One can also obtain the characteristic equation, and the dispersion lines by searching for the poles of the reflection coefficient of the bifilm.[1] To obtain the dispersion lines of the structure, we work in either the complex frequency $\tilde{\omega}$ and real transverse wavevector $\mathrm{Re}[k_x]$ space, or in the real frequency ω and complex wavevector k_x space, and search for the minima of $|\det(L)|$. We use the former solution space for the calculation of mode profiles, field enhancement and mode confinement, and the latter for the calculation of the propagation lengths of the hybrid polaritons. In both scenarios, we first find the minima of $|\det(L)|$ for real frequency ω and $\mathrm{Re}[k_x]$ for the situation where we ignore the losses in both the gold and the ITO layer by using only the real part of their permittivities in the $|\det(L)|$ function. We then use these $\{\omega, \mathrm{Re}[k_x]\}$ solutions as the initial conditions while searching for the minima of $|\det(L)|$ in the $\{\tilde{\omega}, \mathrm{Re}[k_x]\}$ space or the $\{\omega, k_x\}$ space, for which we use the Nelder-Mead method.[7] We use MATLAB for all these calculations, and its built-in function `fminsearch` for the minima search.

Figure S6 compares the dispersion lines (real frequency and wavevector) of bifilm A

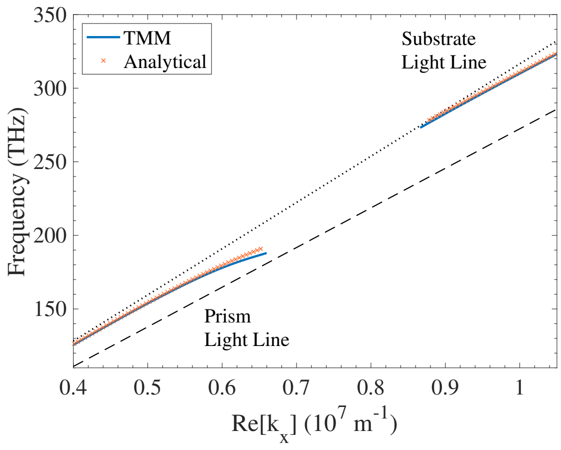

Figure S6: Dispersion line of bifilm A obtained from the TMM reflectance map (blue, solid), and from the analytical dispersion model (red crosses).

calculated by curve-fitting asymmetric Lorentzians to the spectral minima at each Re[k_x] in its TMM reflactance map (blue, solid line), and the analytically calculated dispersion line (red dots). We find a very close agreement in the dispersion lines calculated from the two methods, which verifies our analytical dispersion model, and our subsequent calculations from the model.

S7. The calculation of mode profiles

To calculate the mode profiles of the hybrid polaritons, we need the coefficient vector C, which lies in the null space of the ill-conditioned matrix L. Hence, we first perform a singular value decomposition (SVD) of L at a point $\{\tilde{\omega}, \mathrm{Re}[k_x]\}$ of the dispersion line, which yields a unitary matrix U whose columns are the left singular vectors, a diagonal matrix S whose elements are the singular values of the L, and a unitary matrix V whose columns are the right singular vectors. The column of V that corresponds to the diagonal element in S that has the smallest singular value is the coefficient vector C. We susbtitute these coefficients in the equations (6)-(8) to get the electric field distributions of the hybrid polaritons at each point $\{\tilde{\omega}, \mathrm{Re}[k_x]\}$ on the dispersion line. To calculate the electric field profiles of the standalone samples (gold film on float glass substrate, and the ITO film on the float glass

substrate), we follow the same procedure used for the hybrid modes detailed above, but with the required changes in the characteristic equation, and the coefficient vector C, which now has only 6 elements.

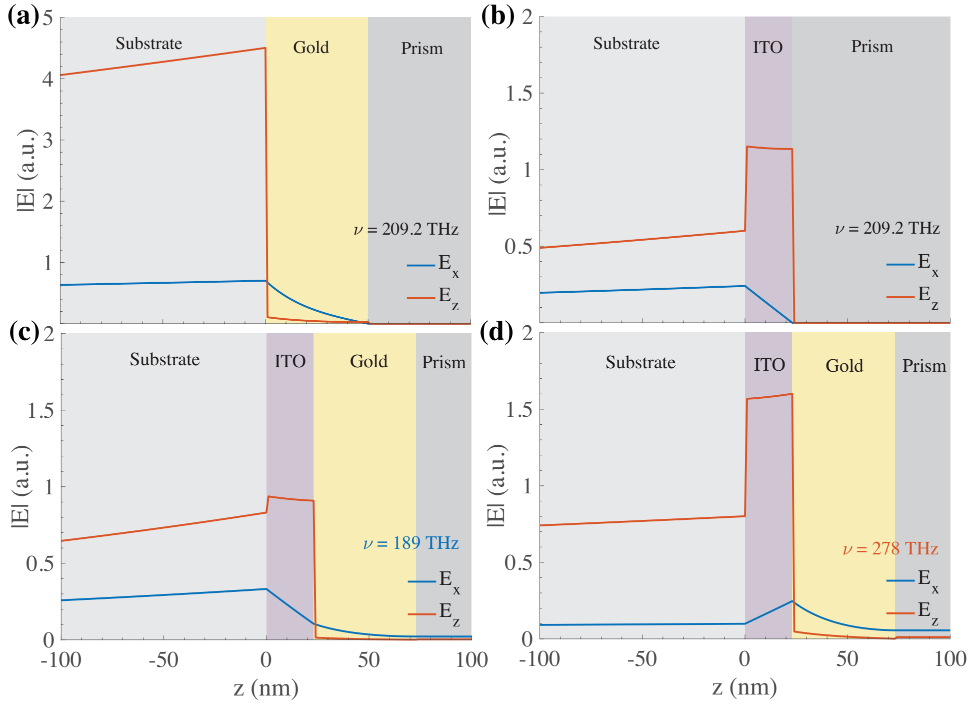

Figure S7: Electric field profiles of the longitudinal "z" (red) and the transverse "x" (blue) components of the (a) standalone LR-SPP mode in a 50 nm gold film, and the (b) standalone ENZ mode in a 23 nm ITO film at their point of degeneracy (where their respective dispersion lines cross). The electric field profiles of the (c) lower and the (d) upper polaritons for bifilm A (50 nm thick gold on 23 nm thick ITO) close to the avoided crossing region.

Figures S7(a) and S7(b) show the electric field profiles of the LR-SPP mode in 50 nm thick gold on float glass, and the ENZ mode in 23 nm thick ITO (with the permittivity shown in Figure S1(a)) on glass, respectively at their point of degeneracy, or where their respective dispersion lines cross. As mentioned previously, the SPP mode is mostly confined at the gold-substrate interface, with a very small longitudinal field amplitude E_z in the gold layer. The ENZ mode, on the other hand, is tightly confined within the ITO film, where E_z is enhanced almost by a factor of three than at the ITO-substrate interface, and is largely constant within the film.

Figures S7(c) and (d) show the electric field profiles of the lower and the upper polariton of

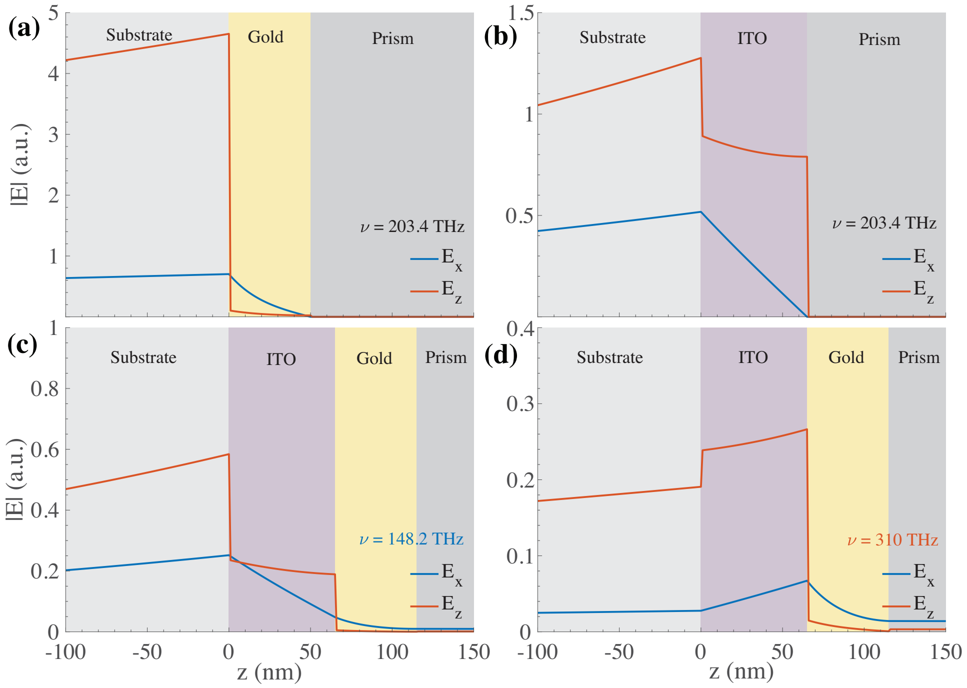

Figure S8: Electric field profiles of the longitudinal "z" (red) and the transverse "x" (blue) components of the (a) standalone LR-SPP mode in a 50 nm gold film, and the (b) standalone ENZ mode in a 65 nm ITO film at their point of degeneracy (where their respective dispersion lines cross). The electric field profiles of the (c) lower and the (d) upper polaritons of the bifilm with 50 nm thick gold on 65 nm thick ITO close to the avoided crossing region.

bifilm A, respectively, close to the avoided crossing region, where both modes are maximally hybridized between the SPP mode and the ENZ mode. Accordingly, we see the characteristic features of both constituent modes in the field profiles of the hybrid modes. Hence, both modes have a large and almost constant E_z in the ITO film. On the other hand, E_x is enhanced along the gold-ITO interface for the upper polariton, and along the ITO-glass interface for the lower polariton. This difference emerges due to the fact that the upper (lower) polariton is formed by a symmetric (anti-symmetric) superposition of the SPP and the ENZ modes.

Figures S8(a) and S8(b) show the electric field profiles of the LR-SPP mode in 50 nm thick gold film on float glass, and the "ENZ" mode in a 65 nm thick ITO film on float glass with the same permittivity as in Figure S7(b), respectively at their point of degeneracy. We see that for this thicker ITO film, the "ENZ" mode starts to resemble the SPP mode so that the fields

are now confined along the ITO-substrate interface, and E_z is no longer strongly enhanced within the ITO film. For the bifilm comprising the 50 nm thick gold on 65 nm thick ITO, the electric field of the hybrid polaritons within the ITO film starts to decouple between the two ITO interfaces, as seen in the electric field profiles of the upper and the lower polariton close to the avoided crossing region shown in Figures S8(c) and S8(d), respectively. Both E_x and E_z for the upper (lower) polariton are enhanced along the gold-ITO (ITO-substrate) interface.

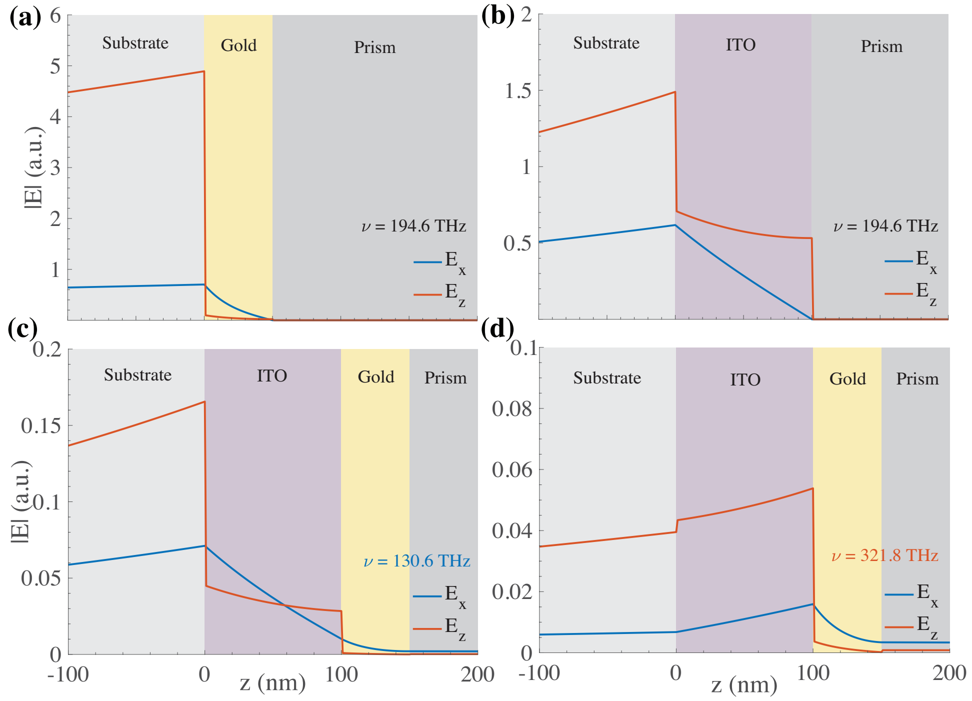

Figure S9: Electric field profiles of the longitudinal "z" (red) and the transverse "x" (blue) components of the (a) standalone LR-SPP mode in a 50 nm gold film, and the (b) standalone ENZ mode in a 100 nm ITO film at their point of degeneracy (where their respective dispersion lines cross). The electric field profiles of the (c) lower and the (d) upper polaritons of the bifilm with 50 nm thick gold on 100 nm thick ITO close to the avoided crossing region.

For even thicker ITO films, the "ENZ" mode becomes even more SPP-like in nature. As shown in Figure S9(b), both E_z and E_x components of the "ENZ" mode for a 100 nm thick ITO film are confined along the ITO-substrate interface. For the bifilm comprising this 100 nm thick ITO film, the hybrid polaritons clearly evolve into two interface polaritons, with the upper polariton confined along the gold-ITO interface, and the lower polariton confined along

the ITO-substrate interface. We can also understand this effect from the behavior of ITO in the spectral range under consideration. ITO behaves like a dielectric for frequencies larger than $\omega_{\mathrm{ENZ,ITO}}$ with a refractive index approaching the float glass substrate for the range of frequencies where the upper polariton exists. Hence, this 100 nm thick ITO layer behaves like a dielectric boundary for the gold film, and leads to a plasmon polariton confined along the gold-ITO interface. For the range of frequencies corresponding to the lower polariton, the ITO response is metal-like. Hence, for these frequencies, the entire bifilm behaves as a thick metal film that supports a long range polariton along the ITO-substrate interface. For such bifilms, the field enhancement within the ITO layer is significantly lower than for the bifilms with a thinner ITO layer. Hence, even though the coupling strength of the SPP and the ENZ modes for such bifilms is strong enough to be classified as being in the ultra-strong coupling region, increasing the thickness of the ITO layer in the bifilm beyond 65 nm does not offer much practical advantage in terms of applications pertaining to accessing the giant nonlinear response of ITO in a guided wave geometry.

S8. The calculation of propagation lengths

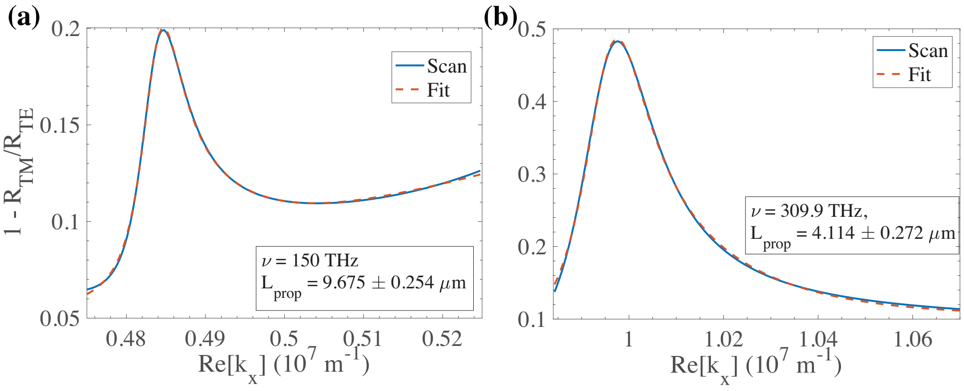

Figure S10: Asymmetric Lorentzian fits (red, dashed) to the extinction scan ($(1-\mathrm{R_{TM}}/\mathrm{R_{TE}})$, blue, solid) for the (a) lower, and the (b) upper polariton. The frequency ν, and the 95 % confidence intervals of the estimated propagation lengths L_{prop} are stated in the inset.

To calculate the propagation lengths of the hybrid polaritons, and the SPP mode from

their reflectance map - both simulated and measured - we perform a nonlinear least-squares curve fit of asymmetric Lorentzian functions of the following form to the extinction ($1 - R_{\text{TM}}/R_{TE}$) wavevector scan at each frequency

$$f_\omega(k_x) = \frac{2C_1/\pi\sigma(k_x)}{1 + [(k_x - k_{x0})/\sigma(k_x)]^2} + C_2 + C_3 k_x, \tag{12}$$

where

$$\sigma(k_x) = \frac{2\sigma_0}{1 + e^{a(k_x - k_{x0})}}. \tag{13}$$

The fitting function is similar to the one assumed for the extracting the decay rates γ_0 from the reflectance maps. The fit parameter k_{x0} calculated from the wavevector scans at various ω produces the dispersion line, albeit with back-bending present where relevant.[8,9] On the other hand, the propagation length L_{prop} of the polariton is given by

$$L_{\text{prop}} = \frac{1}{2\sigma_0}. \tag{14}$$

Figures S10(a) and (b) show a representative example of the fits (red, dashed) to the simulated extinction scans (blue, solid) at two frequencies in the lower, and in the upper polariton branches of bifilm A, respectively. There is a reasonable agreement between the fitted curve, and the actual scan. To calculate L_{prop} of the hybrid polaritons from the analytical dispersion model discussed in section S5, we solve the characteristic equation $|\det(L)| = 0$ in real frequency ω and complex wavevector k_x space. Thus, σ_0 at each frequency is directly obtained from the imaginary part of the k_x solution. We note that this solution does not take into account the radiative losses into the prism, and only take into account the absorption losses within the bifilm.[1]

To calculate L_{prop} of the ENZ mode, we first extract the linewidth γ of the spectral dip from the reflectance maps shown in Figures S2. We then use the following relation from the

supplement in Ref.[10] to calculate the propagation length

$$L_{\text{prop}} = \frac{d_{\text{ITO}}}{4} \frac{\omega^4 + \gamma^2\omega^2}{\omega_p^2 \gamma \omega}. \tag{15}$$

S9. The properties of the upper polariton in bifilms B and C

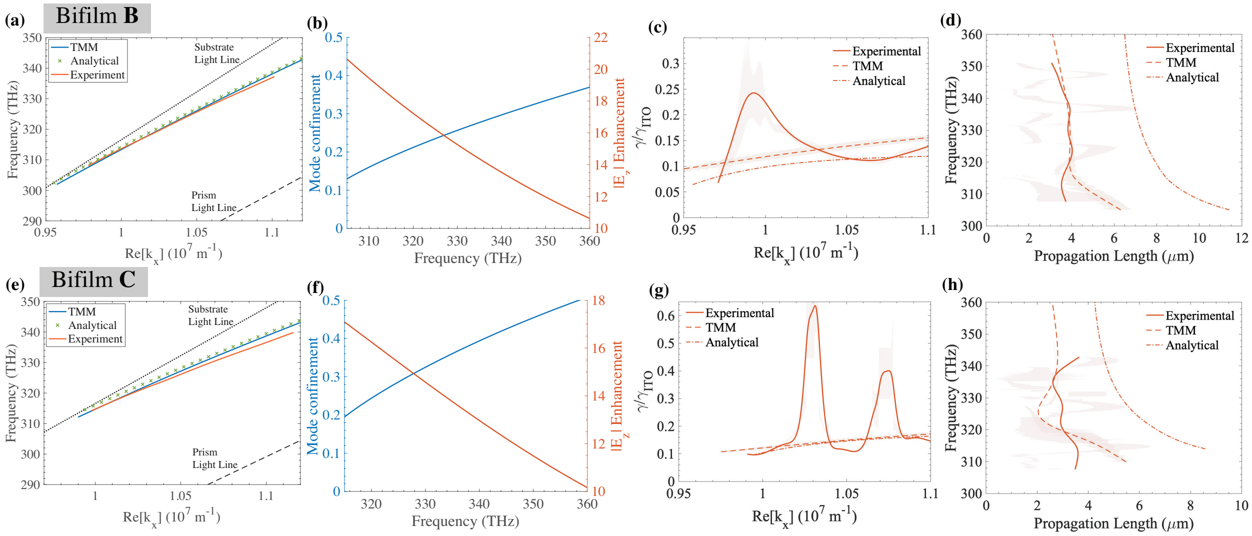

Figure S11: Dispersion lines of the upper polariton of bifilms (a) B and (e) C obtained from TMM simulations (blue, solid), experimental measurements (red, solid), and the analytical model (green crosses). Mode confinement (blue) and the longitudinal field enhancement (red) of the upper polariton in bifilms (b) B and (f) C. The damping γ (normalized to the damping in the Drude permittivity model of the corresponding ITO layer) of the upper polariton in bifilms (c) B and (g) C obtained from the experimental measurements (solid), TMM simulations (dashed), and the analytical model (dot-dashed). The propagation length of the upper polariton in bifilms (d) B and (h) C obtained from the experimental measurements (solid), TMM simulations (dashed), and the analytical model (dot-dashed). The lines representing the simulated, and the measured results in panels (c), (d), (g) and (h) are the smoothened parameters extracted through curve-fitting to the respective datasets to account for fitting errors – and measurement errors in the case of experiment – represented by the red shaded regions around the lines.

Figures S11(a) and (e) show the dispersion lines of the upper polariton in bifilms B and C, respectively. The blue (red) solid lines represent the results from TMM simulations (ex-

perimental measurements) in both panels, and are obtained as before by fitting asymmetric Lorentzians to the corresponding reflectance maps. The green crosses in both panels represent the results from the analytical dispersion model, and are the real part of the complex $\tilde{\omega}$ that minimize the $|\det(L)|$ given in equation (11) at each real wavevector $\mathrm{Re}[k_x]$. The dispersion lines obtained form TMM simulations are in almost perfect agreement with those obtained from the analytical model. The slight differences observed in the experimentally measured dispersion lines with the simulations and the analytical model could be attributed to several factors. First, there could be possible differences in the permittivity values of gold and ITO in the fabricated bifilms and the permittivity values assumed in the simulations, and in the analytical model. Second, the measurement errors, as well as the errors in curve fitting to the measured reflectance maps for the parameter extraction could also contribute to the slight differences.

Figures S11(b) and (f) show the mode confinement (blue, left axis), and the longitudinal field enhancement (red, right axis) within the ITO layer for the upper polariton of bifims B and C, respectively. These quantities are calculated from the mode profiles of the upper polariton (calculated using the method described in section S6) for both bifilms using the definitions of the quantities given in the main text. The upper polariton of bifilm B has slightly larger mode confinement than the upper polariton in bifilm A (Figure 4(e)) at frequencies close to the band edge of bifilm B (around 0.093 and 0.13 for bifilms A and B, respectively). However, the slope of the confinement curve is steeper for bifilm B than for bifilm A, and becomes as large as 0.4 for bifilm B away from the polariton band edge, which is more than thrice the confinement at that particular frequency (350 THz) for the upper polariton in bifilm A. The mode confinement of the upper polariton in bifilm C is even larger than the upper polariton in bifilm B throughout spectral range with a maximum value close to 0.5. This trend is in agreement with the transition of the hybrid polaritons to interface polaritons for larger thicknesses of the ITO layer discussed previously in section S6. At the “bluer” frequencies, where ITO behaves as a dielectric, the (upper polariton) becomes

more confined along the gold-ITO interface for both bifilms B and C. This confinement is even larger for bifilm C where the ENZ mode in the 100 nm-thick-ITO layer is even more LR-SPP-like than the ENZ mode in the 65 nm-thick-ITO layer in bifilm B.

On the other hand, the longitudinal field enhancement of the upper polariton in bifilm A is larger than both bifilms B and C throughout the spectral range under consideration. The maximum field enhancement for the upper polariton in bifilm A $\approx 32\times$ at the band edge of bifilm A, whereas it is $\approx 20\times$ and $\approx 17\times$ at the band edges of bifilms B and C, respectively. A larger field enhancement would be more desirable for enhancing the nonlinear optical response for certain applications. The larger mode confinements of the upper polaritons in bifilms B and C in comparison to the polariton in bifilm A also reflects in their smaller propagation lengths and larger damping. The simulated as well as the measured propagation lengths for the upper polaritons in both bifilms B and C is between 2-4 μm, whereas the corresponding lengths for bifilm A is between 4-8 μm. On the other hand, the simulated and the measured damping rates of the upper polaritons in both bifilms B and C is larger than $0.1\gamma_{\mathrm{ITO}}$, while the damping rate for the upper polariton in bifilm A is lower than $0.1\gamma_{\mathrm{ITO}}$ throughout.

References

1. Novotny, L.; Hecht, B. *Principles of nano-optics*; Cambridge university press, 2012.

2. Stancik, A. L.; Brauns, E. B. A simple asymmetric lineshape for fitting infrared absorption spectra. *Vibrational Spectroscopy* **2008**, *47*, 66–69.

3. Hopfield, J. J. Theory of the contribution of excitons to the complex dielectric constant of crystals. *Phys. Rev.* **1958**, *112*, 1555–1567.

4. Campione, S.; Brener, I.; Marquier, F. Theory of epsilon-near-zero modes in ultrathin films. *Phys. Rev. B* **2015**, *91*, 121408.

5. Todorov, Y.; Andrews, A. M.; Colombelli, R.; De Liberato, S.; Ciuti, C.; Klang, P.; Strasser, G.; Sirtori, C. Ultrastrong light-matter coupling regime with polariton dots. *Phys. Rev. Lett.* **2010**, *105*, 196402.

6. Askenazi, B.; Vasanelli, A.; Delteil, A.; Todorov, Y.; Andreani, L.; Beaudoin, G.; Sagnes, I.; Sirtori, C. Ultra-strong light–matter coupling for designer Reststrahlen band. *New J. Phys.* **2014**, *16*, 043029.

7. Dionne, J. A. Flatland photonics: circumventing diffraction with planar plasmonic architectures. Ph.D. thesis, California Institute of Technology, 2009.

8. Törmä, P.; Barnes, W. L. Strong coupling between surface plasmon polaritons and emitters: a review. *Rep. Prog. Phys.* **2014**, *78*, 013901.

9. Archambault, A.; Teperik, T. V.; Marquier, F.; Greffet, J. J. Surface plasmon Fourier optics. *Phys. Rev. B* **2009**, *79*.

10. Runnerstrom, E. L.; Kelley, K. P.; Folland, T. G.; Nolen, J. R.; Engheta, N.; Caldwell, J. D.; Maria, J. P. Polaritonic Hybrid-Epsilon-near-Zero Modes: Beating the Plasmonic Confinement vs Propagation-Length Trade-Off with Doped Cadmium Oxide Bilayers. *Nano Lett.* **2019**, *19*, 948–957.
